# Atmospheric-Pressure Plasma Jet Induces Apoptosis Involving Mitochondria via Generation of Free Radicals

**DOI:** 10.1371/journal.pone.0028154

**Published:** 2011-11-29

**Authors:** Hak Jun Ahn, Kang Il Kim, Geunyoung Kim, Eunpyo Moon, Sang Sik Yang, Jong-Soo Lee

**Affiliations:** 1 Department of Molecular Science and Technology and Department of Life Science, Ajou University, Suwon, Gyounggi-Do, Korea; ? Department of Electrical and Computer Engineering, Ajou University, Suwon, Gyounggi-Do, Korea; Karolinska Institutet, Sweden

## Abstract

The plasma jet has been proposed as a novel therapeutic method for anticancer treatment. However, its biological effects and mechanism of action remain elusive. Here, we investigated its cell death effects and underlying molecular mechanisms, using air and N_2_ plasma jets from a micro nozzle array. Treatment with air or N_2_ plasma jets caused apoptotic death in human cervical cancer HeLa cells, simultaneously with depolarization of mitochondrial membrane potential. In addition, the plasma jets were able to generate reactive oxygen species (ROS), which function as surrogate apoptotic signals by targeting the mitochondrial membrane potential. Antioxidants or caspase inhibitors ameliorated the apoptotic cell death induced by the air and N_2_ plasma jets, suggesting that the plasma jet may generate ROS as a proapoptotic cue, thus initiating mitochondria-mediated apoptosis. Taken together, our data suggest the potential employment of plasma jets as a novel therapy for cancer.

## Introduction

Apoptosis, one of the major types of cell death, can be modulated by programmed control mechanisms. In contrast to acute traumatic cell death, apoptosis is important not only in the turnover of cells for regeneration in all types of tissues, but also during the normal development, differentiation, and senescence of organisms [1]. In contrast to its significant roles in physiological processes, defective apoptotic processes have been implicated in a variety of diseases, such as atrophy, autoimmunity, neurodegenerative disorders, acquired immune deficiency syndrome, and uncontrolled cell proliferation as found in cancer [2].

Mitochondria are well known as the primary coordinators of apoptotic processes to control the intrinsic apoptotic pathway. The process of apoptosis is controlled by a diverse range of extrinsic and intrinsic cell signals. Several intracellular signals can converge on mitochondria to induce mitochondrial swelling or increase mitochondrial membrane permeability (MMP), which causes the dissipation of mitochondrial transmembrane potential (Δψm) and the release of proapoptotic factors, including cytochrome *c*. Mitochondrial outer membrane proteins, which are regulated by the anti- and pro-apoptotic members of the Bcl-2 family, and proteins released from mitochondria, lead to activation of caspases and subsequent cell death [3]. The parameters of mitochondrial physiology, including the loss of mitochondrial transmembrane potential or swelling, have been shown to be hallmarks of mitochondria-dependent apoptosis [4].

There is growing evidence that the redox environment of a cell is able to control apoptosis. Free radicals can induce apoptosis by dissipation of mitochondrial transmembrane potential [5]. The major free radicals are reactive oxygen species (ROS) and nitric oxide (NO), which are derived from oxygen and nitrogen under reducing conditions [6]. Free radicals play an important role in a number of biological processes, such as the intracellular killing of bacteria by phagocytic cells. They have also been implicated in cellular redox signalling. However, because of their reactivity, excessive amounts of free radicals can lead to cell damage and death. ROS are removed by antioxidant enzymes including catalase (CAT) and superoxide dismutase (SOD). An imbalance of the redox milieu develops and leads to accumulation of ROS, resulting in oxidative stress [7]. Another free radical, NO, is an important cellular messenger molecule involved in many physiological and pathological processes, in mammals. However, direct cellular damage results from increased levels of NO. NO interacts with ROS in a variety of ways, either as a crucial partner in regulating the redox status of cells, determining cell fate, or in signalling in response to a number of physiological and stress conditions.

Recently, we and others have reported that atmospheric-pressure plasma [8]-[12] and nitrogen plasma jets from a micro nozzle array induce apoptosis of cancer cells by generating DNA damage [13]. However, the molecular mechanism by which plasma induces apoptosis and what signal(s) stimulate the plasma-induced apoptosis remain unclear. The atmospheric-pressure plasma jet can generate ROS such as ozone, atomic oxygen, superoxide, peroxide, and hydroxyl radicals but it is not known whether the ROS generated by plasma jets can mediate apoptosis of mammalian cancer cells or whether the plasma-induced apoptotic processes involve mitochondria. In this report, we show that micro plasma jet nozzle array generate ROS that may act as an apoptotic stimulus by inducing altered MMP in human cervical carcinoma HeLa cells. However, free radical scavengers or caspase inhibitors can mitigate the plasma-induced apoptotic effects. Taken together, our findings indicate that free radicals generated by plasma play a role in plasma-induced apoptosis by acting as an intrinsic signal to initiate apoptosis via the involvement of the mitochondria.

## Results and Discussion

### Plasma jet from micro nozzle array

**Figure 1:**
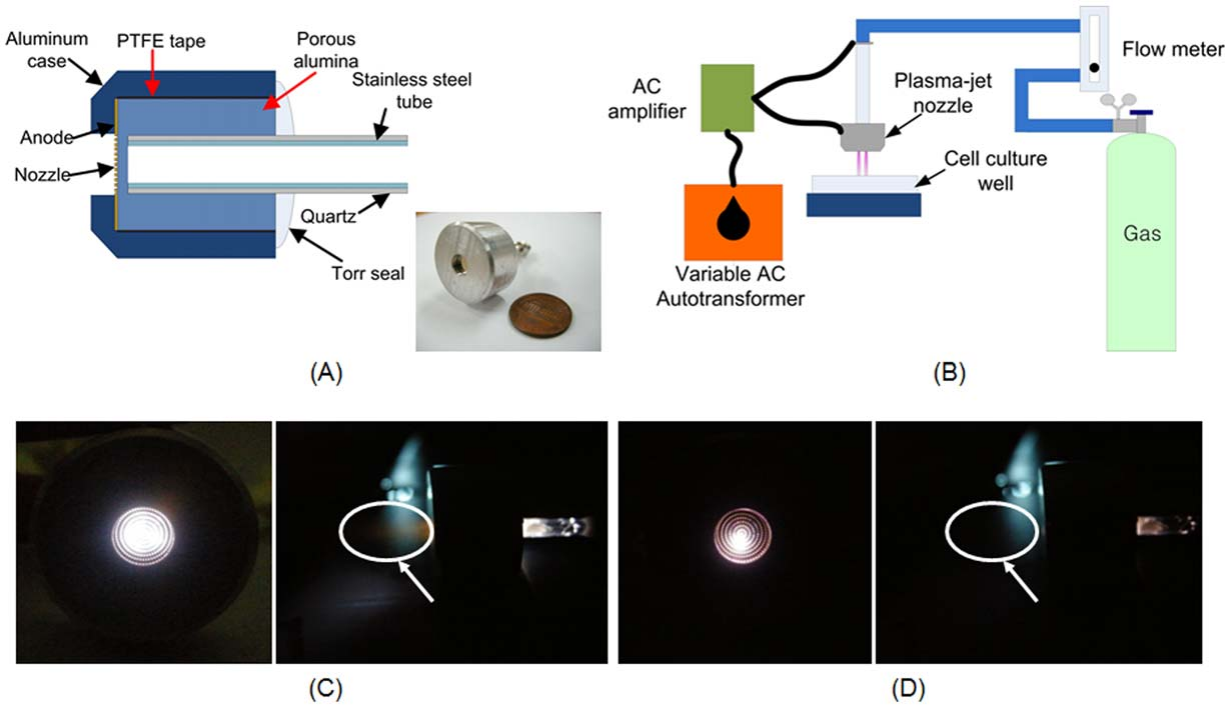
(A) Schematic structure of a plasma jet nozzle array. The inset shows a photograph of the fabricated plasma jet nozzle device. (B) A schematic view of the experimental setup for cell treatment. (C)–(D) Photographic images of the microplasma jet nozzle during discharges when (C) N_2_ and (D) air are used. The left side shows a front view, while the right side shows a side view. The plasma jet is indicated as circles and arrows.

**Figure 2:**
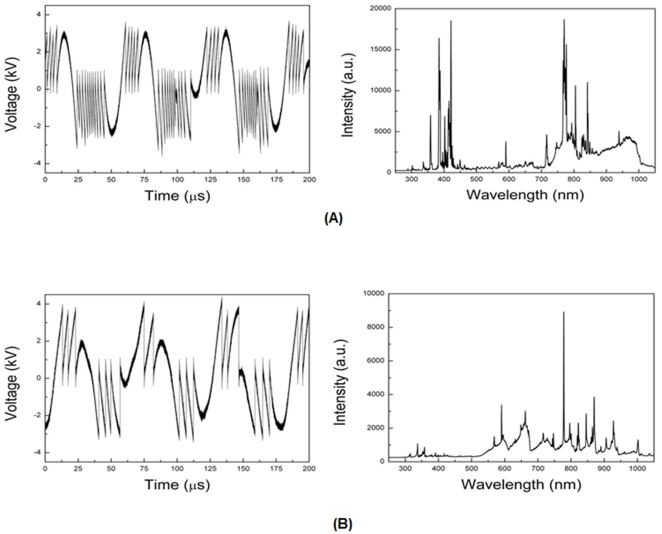
The measured voltage waveforms and optical emission spectra of (A) N_2_ and (B) air during discharges at an input voltage of 20 kV_p-p_.

The plasma jet used in this study emanated from a micro-nozzle array (Fig. 1(C)–(D)). The air plasma jet spouts out into the open air through the anode and exhibits characteristics different from those of the nitrogen plasma jet [14]. In order to verify the characteristics of the plasma jet, we measured the voltage and performed optical emission spectroscopy (OES) during the discharges. The voltage generated by a discharge through the porous alumina fluctuated significantly (Fig. 2(A)). The electrical characteristics of the plasma jet device using porous alumina is reported that the pulse voltage dropped to nearly zero in conjunction with sharp current pulses [15]. As shown in Fig. 2(A), N_2_ plasma generated a heavier saw-tooth waveform than air plasma during the same duration, demonstrating that the density of the N_2_ plasma jet was higher than that of the air plasma jet.

Generally, the atmospheric-pressure plasma jet can produce chemically active species. To identify the various excited plasma species generated by the plasma jet, OES was carried out for a wide range of wavelengths, 250–1050 nm. The optical emission of N_2_ was compared with that of air plasma at 10 L/min. The spectrum of the N_2_ plasma was dominated by the bands of N_2_ molecules (second positive system (SPS) for λ in the range 290–500 nm and first positive system (FPS) for λ in the range 700–900 nm). The emission spectrum of the air plasma was dominated by the presence of excited oxygen atoms at 777.1 and 844 nm, and molecules in the range of 500–600 nm, containing N_2_ SPS and FPS.

### Analysis of plasma jet-induced cell death, using annexin V-FITC and PI staining

**Figure 3:**
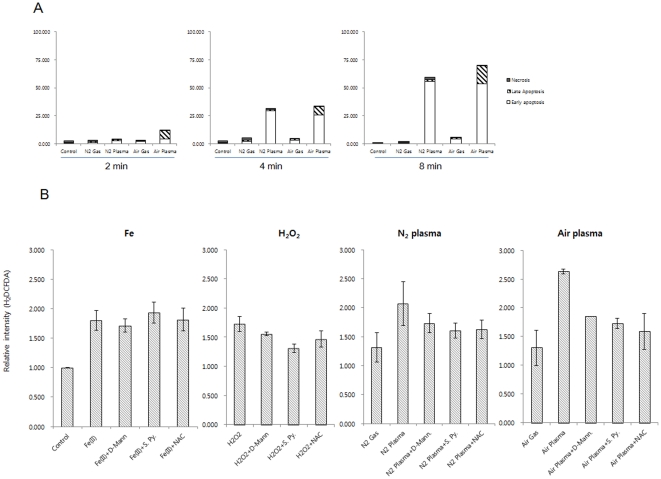
Plasma induces apoptosis via generation of ROS. (A) Apoptosis in HeLa cells, a human cervical carcinoma cell line, was analysed by flow cytometry, following staining with anti-Annexin V-FITC and PI. HeLa cells were treated with a N_2_ or air plasma jet for 2–8 min as indicated, and incubated further for 24 hr. Cells in early apoptosis are stained with Annexin V antibody only, while cells at the late apoptotic stage are stained with both annexin V antibody and PI. Necrotic cells were stained with only PI. Data are shown as the mean ± s.e.m. (*n* = 8). (B) ROS generation was monitored by flow cytometry. Cells were incubated with 2.5 µM H_2_DCFDA, harvested, and within 1 hr of treating cells with a N_2_ or air plasma jet for 5 min, the level of ROS was evaluated by measuring the emission of DFC. Non-treated (-), Ferrous (II) perchlorate- (Fe), and H_2_O_2_- treated cells (H_2_O_2_) were employed as controls. As antioxidants, D-mannitol (D-Mann), sodium pyruvate (S Py.), or N-Acetyl-Cysteine (NAC) was used. Data are shown as the mean ± s.e.m. (*n* = 13).

To better understand the biological effects and application of plasma jets, we investigated the cell death induced by plasma jets. A plasma jet was applied directly to HeLa cancer cells, which were then stained with FITC dye conjugated annexin V antibody or PI, and analysed by flow cytometry. Dot plots of 4 quadrants logarithmically scaled were created to represent the fluorescence levels of FITC-labelled annexin V (FITC-A) and PI (PI-A). The flow cytometry analysis revealed that N_2_ and air plasma jets augmented the number of early apoptotic cells (annexin V^+^/PI^−^), late apoptotic cells (annexin V^+^/PI^+^), but rarely necrotic cells (annexin V^−^/PI^+^), compared with untreated HeLa cells (Fig. S1). Next, we examined apoptosis in cells exposed to the plasma jet for a variety of treatment durations, to determine whether plasma jets induce apoptosis in a duration-dependent manner. Cells were treated with a plasma jet for 2–8 min, then stained and analysed as described above (Fig. 3(A)). Treatment of HeLa cells with N_2_ and air plasma for 2 min had little apoptotic effect on HeLa cells. In contrast, treatment with plasma jets for 5 min increased apoptosis by approximately 32% (early, 29.72±1.11% and late, 0.91±0.35%) (N_2_) and 34% (early: 25.80±1.76% and late: 7.58±0.54%) (air). Higher apoptotic effects of 60% (early, 55.69±11.36% and late, 1.70±0.35%) (N_2_ plasma) and 70% (early, 53.80±12.04% and late, 15.80±3.54 %) (air plasma) were achieved by treatment for 8 min. On the contrary, the plasma induced marginally necrosis (N_2_ plasma, 0.1–2.0% or air plasma, 0.2–0.3 %) (Fig. S1). Together, these data suggest that N_2_ and air plasma jets induce largely apoptotic cell death in a dose-dependent manner.

### Depolarization of mitochondria membrane potential and generation of ROS are induced by N_2_ and air plasma jet

ROS are known as triggers of the intrinsic apoptotic cascade via interaction with proteins of the mitochondrial permeability transition complex [16]. Components of the permeability transition pore (PTP), including the voltage-dependent anion channel (VDAC), are targets of ROS and oxidative modifications of PTP proteins can significantly affect mitochondrial anion fluxes [17]. Indeed, a mere transient increase in mitochondrial membrane hyperpolarization following exposure to H_2_O_2_ initiates the collapse of the mitochondrial transmembrane potential (Δψm) [18], leading to mitochondrial translocation of Bax and Bad and cytochrome c release [19]. Based on reports that atmospheric pressure plasma jets can generate ROS [20], [21], we speculated on the possibility that plasma jets generate ROS in mammalian cells, and consequently the generated ROS may directly link to induction of apoptosis. As shown in Fig. 3(B) and Fig. S2, the level of ROS increased by approximately 2-fold and 2.6-fold in HeLa cells treated with N_2_ and air plasma jets, respectively, compared with untreated cells.

**Figure 4:**
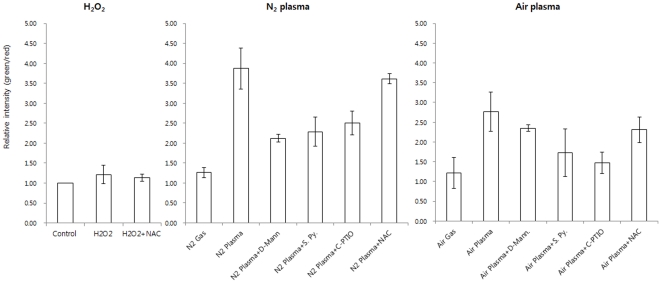
Mitochondrial transmembrane potential (MMP) was measured by flow cytometry, following staining with JC-1 dye. Cells were treated with ROS scavenging agents, d-Mannitol, sodium pyruvate, carboxyl-PTIO or N-Acetyl-cystein (NAC) for 1 hr prior to treatment with a plasma jet. After N_2_ or air plasma jet treatment, cells were incubated for a further 12 hr before harvesting. After harvest, the cells were washed 3 times with PBS and stained with 2.5 µM JC-1 dye for 30 min. Green fluorescence monitoring depolarization of mitochondrial membrane potential (Δψm) of the untreated cells was arbitrarily set to a value of 1. Data are shown as the mean ± s.e.m. (*n* = 7).

Next, we evaluated depolarization of the mitochondrial membrane potential (Δψm) using JC-1 staining following treatment with N_2_ and air plasma jets. Mitochondrial membrane depolarization is an early event of apoptosis that increases mitochondrial membrane permeability, thereby facilitating the release of proapoptotic factors, including cytochrome c and AIF into the cytosol [22]. The mitochondria-specific probe JC-1 is a lipophilic cationic fluorescence dye with a dual emission wavelength. Increased mitochondrial membrane potential has been shown to increase JC-1 fluorescence at 530 nm (FITC-A), which corresponds to its monomeric form, and to reduce JC-1 fluorescence at 590 nm (PI-A), which corresponds to its dimeric form. Fluorescence intensity of JC-1 at 530 nm (FITC-A) was elevated by 3.7-fold and 2.8-fold in the N_2_ and air plasma jet-treated HeLa cells, respectively, compared with non-treated control (Fig. 4 and Fig. S3). The fluorescence intensity of JC-1 at 590 nm (PI-A) was reduced to 1.7-fold and 1.8-fold following treatment with N_2_ and air plasma jet, respectively. These data indicate that plasma jet treatment can induce depolarization of the mitochondria membrane potential, causing increased mitochondrial transmembrane permeability and potential release of proapoptotic factors.

### Antioxidants alleviate apoptotic cell death following N_2_ and air plasma jet treatment

When cells are exposed to a diversity of stressors such as UV irradiation, drugs, hypoxia, chemicals, and many others, ROS are generated and can assault intracellular organelles and membranes, proteins, DNA, and lipids. Cells are equipped with a variety of defence mechanisms, including scavenging enzymes such as catalase and superoxide dismutase (SOD), repair machinery, and regeneration pathways. However, cellular damages can be too severe for cells to adapt or survive, and so cause cell death.

**Figure 5:**
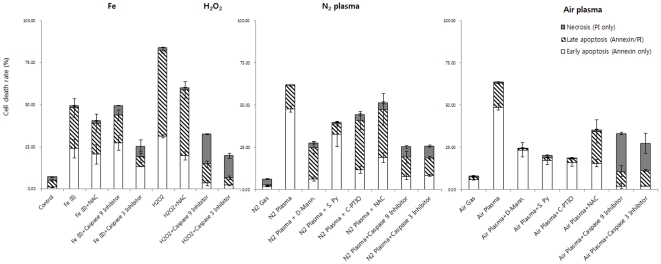
Scavenging agents are able to alleviate the apoptotic effect induced by N_2_ and air plasma exposure in HeLa cells. Cells were treated with D-Mannitol, sodium pyruvate, carboxyl-PTIO or N-Acetyl-cystein (NAC) for 1 hr prior to treatment with an air and N_2_ plasma jet. HeLa cells were exposed to the air and N_2_ plasma jet, immediately after treatment with caspase 3 or 9 inhibitor. Data are shown as the mean ± s.e.m. (*n* = 10). Values were shown in Fig S4.

We tested whether the ROS generated by plasma jets are implicated in plasma-mediated apoptosis. To this end, we assessed the plasma jet-induced apoptosis in cells treated with ROS scavenging agents. Mannitol and sodium pyruvate are well known scavengers of hydroxyl groups (HO^•^) and hydrogen peroxide (H_2_O_2_), respectively. Carboxy-PTIO is an effective scavenger of nitric oxide (NO^•^) [23] and N-acetyl-Cystein (NAC) is a well-known thiol antioxidant [24]. In combination with the plasma jet, HeLa cells were treated with D-mannitol, sodium pyruvate, carboxyl-PTIO or N-acetyl-cysteine. In control experiments, mannitol, sodium pyruvate, carboxyl-PTIO or NAC alone, barely affected the growth or death of HeLa cells (data not shown). In combination with treatment with the antioxidant mannitol, apoptosis induced by the plasma jet was alleviated to 27.38±5.30% (early, 5.99±1.18% and late, 18.75±3.69 %) (N_2_) and 24.75±4.48% (early, 23.39±4.30% and late, 0.99±0.18 %) (air), compared with apoptotic cell death in the cells treated with the plasma jet alone (61.79±2.36 %, (N_2_) and 63.70±2.55%, (air)) (Fig. 5 and Fig. S4). In a similar fashion, sodium pyruvate reduced the plasma-induced apoptosis to 39.60±6.36% (early, 32.63±7.26% and late, 6.23±1.39 %) (N_2_) and 20.28±3.16% (early, 17.38±2.89% and late, 1.62±0.27 %) (air) (Fig. 5 and Fig. S4). In addition, carboxy-PTIO and NAC attenuated the apoptotic effect by air plasma jet to 18.86±2.90%% (early, 16.15±2.57% and late, 2.29±0.36 %) and 35.44±9.09% (early, 15.66±2.13% and late, 18.75±6.84 %), respectively (Fig. 5 and Fig. S4). Also, the cell death induced by N_2_ plasma jet was partially mitigated by carboxy-PTIO to 44.27±4.86% or by NAC to 47.20±2.36% (Fig. 5 and Fig. S4). These findings suggest that removal of ROS can abrogate plasma-induced cell death and that ROS mediate the plasma jet-induced apoptosis. Furthermore, the abrogation effect of carboxy-PTIO in the air plasma jet treated cells was higher than in the N_2_ plasma jet treated cells (Fig. 5). On the basis of our observations that ROS scavengers exhibited differential abrogation of apoptosis in plasma jet-treated cells, we considered the possibility that air and N_2_ plasma jet may generate different ROS using OES (Fig. 1(C)). We characterized the dominant peak spectra of air and N_2_-plasma jets. The dominant peaks of air-plasma jet spectra was mainly seen in the infra-red (IR) spectrum, while peaks of N_2_ plasma were seen in the UV and IR regions (Fig. 1(D)). If this is the case, we can speculate that different plasma sources might generate different types and compositions of ROS.

### Caspase inhibitors can mitigate apoptotic cell death following N_2_ and air plasma jet treatment

The caspases are one of the main executors of the apoptotic process. Induction of apoptosis typically results in the activation of caspase cascade. The mitochondria are key regulators of the caspase cascade, through activation of an initiator caspase 9 and then of caspase 3. Aside from mitochondria-dependent cascade, induction of apoptosis via activation of death receptors leads to the activation of the initiator caspase 8 or 10, and then caspase 3. To further potentiate that the plasma induces mitochondria-mediated apoptosis, we tested whether caspase 3 and 9 inhibitors can alleviate the plasma-induced apoptosis. Overall, the caspase 3 and 9 inhibitors mitigated the plasma-induced cell death approximately by half (approximately 25% from 61.8% (N_2_) and approximately 30% from 63.7% (air)) (Fig. 5 and Fig. S4). However, the caspase 8 inhibitor had little effect on the plasma-induced apoptosis (data not shown), suggesting that the plasma-induced apoptosis is not mediated through activation of death receptors. Notably, the caspase 3 and 9 inhibitors markedly reduced apoptosis in cells treated with the plasmas, indicating that mitochondria can mediate the plasma-induced apoptotic process (Figure 5 and Fig. S4). Consistently, the caspase 3 and 9 inhibitors abrogated the air plasma-induced early apoptosis (Figure 5 and Fig. S4). Together, these caspase inhibitor effects suggest that the plasma-induced apoptosis is mediated through mitochondria-dependent pathway.

In contrast to the apoptotic cell death, the caspase 3 and 9 inhibitors caused increased necrosis in the plasma-treated cells (Figure 5 and Fig. S4). In a similar manner, the caspase 3 and 9 inhibitors induced necrotic cell death in cells treated with hydrogen peroxide (H_2_O_2_) (Figure 5 and Fig. S4). H_2_O_2_ is known to induce both apoptosis and necrosis [25]. Also, it is reported that H_2_O_2_-treated cells have favourably undergone necrotic cell death rather than mitochondrial-dependent apoptotic cell death in the presence of caspase inhibitors [26]. In this regard, it is possible that the plasma-treated cells may primarily undergo necrotic cell death when the mitochondrial-dependent apoptotic pathway is blocked. Also, caspase 3 and 9 inhibitors could not completely abrogate the plasma-induced cell death, suggesting that the plasma-induced cell death may be mediated partially by mitochondria-dependent pathway. Despite these remaining issues to be resolved in further studies, data presented here strongly indicate that the N_2_ and air plasmas may activate intrinsic proapoptotic pathway and this mitochondria-mediated apoptosis contribute at least in part to the plasma-induced cell death.

In conclusion, we report that treatment with N_2_ and air plasma jet induced apoptotic cell death via ROS generation and dysfunction of mitochondria in the human cervical carcinoma cell line, HeLa, for the first time. Cotreatment of cells with scavengers of ROS mitigated the apoptotic effect of the plasma jet, suggesting that the apoptotic effects of the plasma jet may be mediated by ROS. Also, the alleviating effects of caspase 3 and 9 inhibitors on the plasma-induced cell death further potentiate the involvement of mitochondria in apoptosis. We and others have previously shown that plasma jets may be effective in the treatment of cancers by promoting apoptosis accompanied by DNA damage [27]. Importantly, air and N_2_ plasma jet may be a potent treatment against human cancers. Although additional animal and preclinical studies are required to evaluate the clinical effectiveness of plasma jets, it appears that the plasma jet may have important clinical applications. Further efforts to develop an efficient clinical device and source of plasma jets for cancer treatment are necessary as well. In addition, combinatory treatment of plasma jets with commonly used anticancer drugs or radiotherapy may partially or temporally improve the clinical course by sensitizing resistant or recurrent cancer cells. As such, we suggest that the plasma jet be extensively explored to develop novel cancer therapeutics. Our study implies that application of the plasma jet may provide a potential therapy for cancer.

## Materials and Methods

### Structure of the micro plasma jet nozzle array and plasma discharge system

The microplasma jet nozzle contained a thin nickel anode, a porous alumina insulator, a stainless steel cathode, and an aluminium case. Fig. 1(A) shows a cross-sectional schematic view of the plasma jet device. A Ti/Au film was deposited on a silicon wafer as a seed layer for electroplating. A THB-151N photoresist (JSR, Japan) was patterned for the electroplating mould. A simple electrolyte containing 450 g/L of nickel sulphamate [Ni(NH_2_SO_3_)_2_•4H_2_O] and 30 g/L of boric acid was used to carry out low-stress nickel electroplating. To improve the properties of the electroplated nickel, 5 g/L dodecyl sulphate sodium salt [C_12_H_25_O_4_SNa] was added to the electrolyte. The wafer was electroplated at 50 mA/cm^2^ in this electrolyte for 80 h to a thickness of approximately 80 µm. After the nickel electroplating, the substrate was polished for planarization. The fabricated anode had 210 holes, each having a diameter of 100 µm and a depth of 60 µm. The porous alumina was porous enough to allow gas to pass through [15]. The porous alumina had a porosity of approximately 30 vol% and an average pore diameter of 100 µm. The thickness of the insulator was 1 mm, and the diameter of the cathode was 1.5 mm. For safety, the stainless steel cathode was placed in a quartz tube during the experiments. The inset of Fig. 1(A) shows a photographic image of the fabricated device.

Fig. 1(B) shows the setup for the cell experiments. The discharge experiment was performed at atmospheric pressure with N_2_ and air gas. The AC power supply was a commercial transformer for neon light operated at 20 kHz. An AC voltage of 20 kV_p-p_ and a flow rate of 10 L/min were used in the experiments.

### Cell culture and treatments of cells with plasma jet

Human cervical cancer HeLa cells (ATCC CCL-2) were cultured in DMEM (WelGENE, Korea), supplemented with 10% FBS (Hyclone, Logan, NY) and 500 U/mL penicillin, 200 µg/mL streptomycin and 0.5 µg/mL amphotericin B (WelGENE, Korea) at 37°C in a humidified atmosphere of 5% CO_2_ for 24 h. Cells grown to 90% confluency were exposed to a N_2_ or air plasma jet from the device located 3.8 cm apart, for 2, 5, 6, and 8 min. Immediately after 2 mM hydrogen peroxide (H_2_O_2_), 2 mM ferrous (II) perchlorate (Sigma-Aldrich, Saint Louis, MO) or plasma treatment, the cells were stained with 5 µM H_2_DCFDA, to detect ROS generation. Some plasma-treated cells were further incubated for 12–24 h to measure cell death. After 24 h incubation, cells were harvested and stained with 2.5 µM JC-1 (Molecular Probes, Eugene, OR) to analyse the depolarization of the mitochondria transmembrane potential (Δψm). To detect plasma-induced apoptosis, cells were stained with FITC conjugated annexin V antibody or propidium iodide (PI, BD Bioscience, San Jose, CA). Following staining, cells were analyzed using flow cytometry (BD FACSAria™ III, BD Bioscience, San Jose, CA). The ROS scavenging agents, 50 mM d-mannitol, 10 mM sodium pyruvate (Sigma-Aldrich, Saint Louis, MO), 100 µM carboxy-PTIO (Molecular Probes, Eugene, OR) or 4 mM N-Acetyl-Cysteine (Sigma-Aldrich, Saint Louis, MO) was added, 1 hr before cells were treated with plasma jets, Cells were exposed to a N_2_ or air plasma jet, immediately after adding 20 µM of caspase 3, 8, or 9 inhibitor (Promega, Madison, WI).

## Supporting Information

Figure S1 **Apoptosis in HeLa cells, a human cervical carcinoma cell line, as analysed by flow cytometry.** Cells were treated with N_2_ or air plasma jets for 2, 5, or 8 min and then incubated further for 24 hr. After harvesting, cells were stained with anti-annexin V-FITC and PI and analysed by flow cytometry.
(TIF)

Figure S2 **ROS generation was monitored using H_2_DCFDA.** Cells were stained with H_2_DCFDA, within 1 hr after treatment with N_2_ and air plasma jet for 5 min, then analysed by flow cytometry. The level of ROS generation increased in the N_2_ (2.07±0.38) and air (2.63±0.04) plasma jet-treated cells, compared with the control (1.17±0.02), H_2_O_2_ treated (1.73±0.12), N_2_ gas treated (1.32±0.25), or air gas treated (1.31±0.31) cells. Data are shown as the mean ± s.e.m, which is mean of green fluorescent intensity.
(TIF)

Figure S3 **Mitochondrial transmembrane potential (MMP) was measured with JC-1 dye.** Cells were treated with N_2_ or air plasma jets for 5 min and then incubated for 12 h. After harvesting, the cells were stained with 2.5 µM JC-1 for 30 min, and analysed by flow cytometry. N_2_ and air plasma jet-treatment reduced MMP (6.45±0.16 and 5.88±1.78, ratio of green fluorescence to red fluorescence intensity), in comparison to that in control (1.79±0.74), H_2_O_2_-treated (1.89±0.46), and gas-treated (1.83±0.58) cells. Images were analysed to detect relative green to red fluorescence, using the Zeiss Axioskop 2 microscope (Carl Ziess, Oberkochen, Germany).
(TIF)

Figure S4 **Antioxidants are able to alleviate the apoptotic effect induced by N_2_ and air plasma exposure.** Cells were treated with D-Mannitol (D-Mann), sodium pyruvate (S Py), carboxyl-PTIO (C-PTIO) or N-Acetyl-cystein (NAC), 1 hr prior to treatment with an air and N_2_ plasma jet. Cells were exposed to the air and N_2_ plasma jet, immediately after treatment with caspase 3 or 9 inhibitor. Cells were treated with N_2_ or air plasma jets for 5 min and then incubated further for 24 hr. After harvesting, cells were stained with anti-annexin V-FITC and PI and analysed by flow cytometry. Population (%) of apoptotic and necrotic cells are shown (bottom table).
(TIF)
