# Supplementary figures and images for: Atmospheric-Pressure Plasma Jet Induces Apoptosis Involving Mitochondria via Generation of Free Radicals
Source: PLoS One. 2011 Nov 29;6(11):e28154. doi: 10.1371/journal.pone.0028154 (PMC3226649; doi:10.1371/journal.pone.0028154)

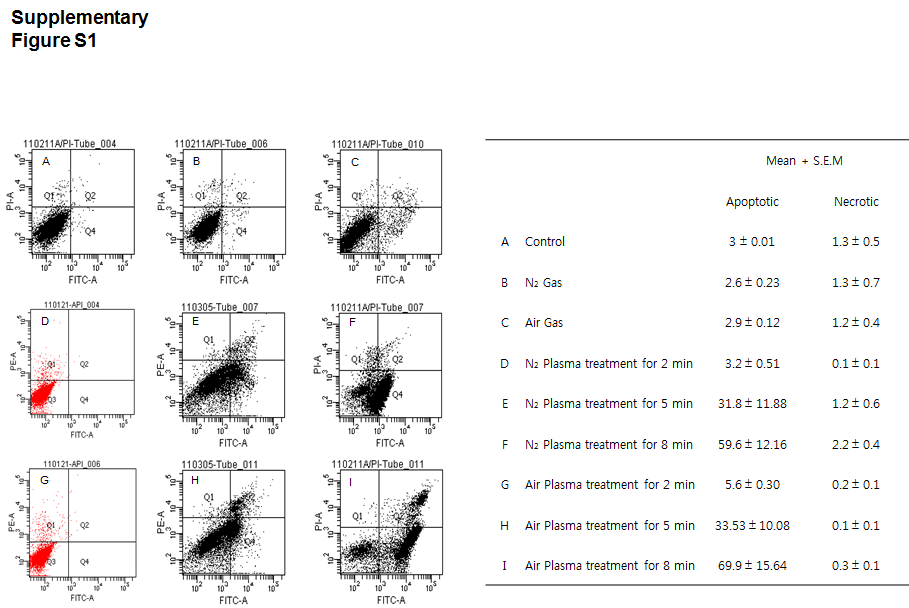

Supplement: Figure S1 — Apoptosis in HeLa cells, a human cervical carcinoma cell line, as analysed by flow cytometry. Cells were treated with N2 or air plasma jets for 2, 5, or 8 min and then incubated further for 24 hr. After harvesting, cells were stained with anti-annexin V-FITC and PI and analysed by flow cytometry. (TIF) [file pone.0028154.s001.tif]

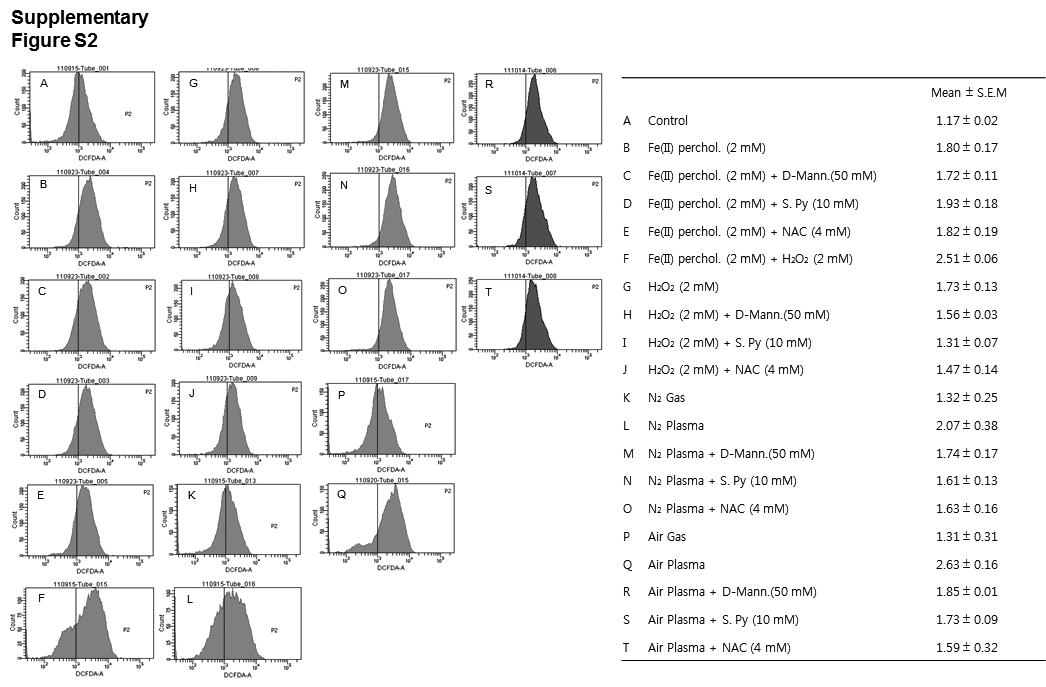

Supplement: Figure S2 — ROS generation was monitored using H2DCFDA. Cells were stained with H2DCFDA, within 1 hr after treatment with N2 and air plasma jet for 5 min, then analysed by flow cytometry. The level of ROS generation increased in the N2 (2.07±0.38) and air (2.63±0.04) plasma jet-treated cells, compared with the control (1.17±0.02), H2O2 treated (1.73±0.12), N2 gas treated (1.32±0.25), or air gas treated (1.31±0.31) cells. Data are shown as the mean ± s.e.m, which is mean of green fluorescent intensity. (TIF) [file pone.0028154.s002.tif]

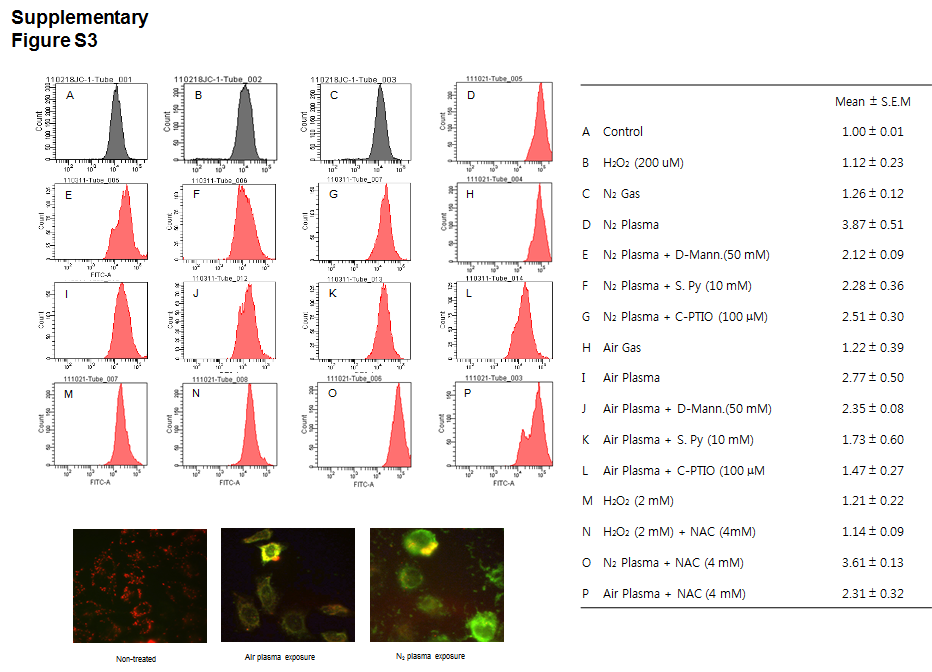

Supplement: Figure S3 — Mitochondrial transmembrane potential (MMP) was measured with JC-1 dye. Cells were treated with N2 or air plasma jets for 5 min and then incubated for 12 h. After harvesting, the cells were stained with 2.5 µM JC-1 for 30 min, and analysed by flow cytometry. N2 and air plasma jet-treatment reduced MMP (6.45±0.16 and 5.88±1.78, ratio of green fluorescence to red fluorescence intensity), in comparison to that in control (1.79±0.74), H2O2-treated (1.89±0.46), and gas-treated (1.83±0.58) cells. Images were analysed to detect relative green to red fluorescence, using the Zeiss Axioskop 2 microscope (Carl Ziess, Oberkochen, Germany). (TIF) [file pone.0028154.s003.tif]

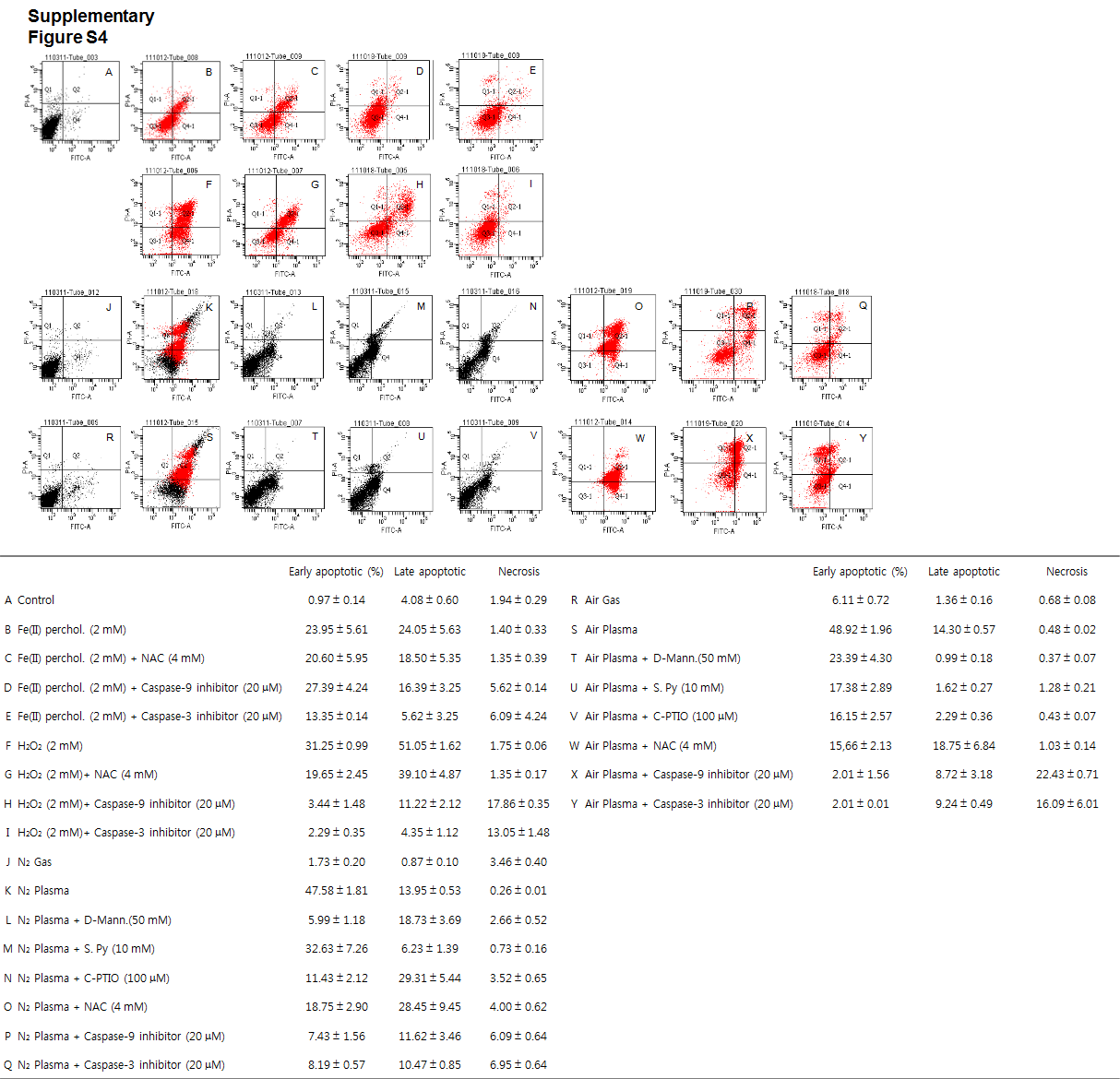

Supplement: Figure S4 — Antioxidants are able to alleviate the apoptotic effect induced by N2 and air plasma exposure. Cells were treated with D-Mannitol (D-Mann), sodium pyruvate (S Py), carboxyl-PTIO (C-PTIO) or N-Acetyl-cystein (NAC), 1 hr prior to treatment with an air and N2 plasma jet. Cells were exposed to the air and N2 plasma jet, immediately after treatment with caspase 3 or 9 inhibitor. Cells were treated with N2 or air plasma jets for 5 min and then incubated further for 24 hr. After harvesting, cells were stained with anti-annexin V-FITC and PI and analysed by flow cytometry. Population (%) of apoptotic and necrotic cells are shown (bottom table). (TIF) [file pone.0028154.s004.tif]
